# Overall and cause-specific mortality in HIV-positive subjects compared to the general population

**DOI:** 10.7448/IAS.17.4.19711

**Published:** 2014-11-02

**Authors:** Belen Alejos, Victoria Hernando, Jose López-Aldeguer, Ferrán Segura, José Antonio Oteo, Rafael Rubio, Arantza Sanvisens, Paz Sobrino, Julia del Amo, Cohort Coris

**Affiliations:** 1 National Center of Epidemiology, Institute of Health Carlos III, Madrid, Spain; 2 CoRIS cohort, Hospital La Fe, Valencia, Spain; 3 CoRIS cohort, Hospital Parc Tauli, Sabadell, Spain; 4 CoRIS cohort, Hospital San Pedro, Logroño, Spain; 5 CoRIS cohort, Hospital Doce de Octubre, Madrid, Spain; 6 CoRIS cohort, Germans Trias i Pujol, Badalona, Spain; 7 CoRIS cohort, Institute of Health Carlos III, Madrid, Spain

## Abstract

**Introduction:**

Emerging non-AIDS related causes of death have been observed in HIV-positive subjects in industrialized countries. We aimed to analyze overall and cause-specific excess of mortality of HIV-positive patients compared to the general population and to assess the effect of prognostic factors.

**Material and Methods:**

We used generalized linear models with Poisson error structure to estimate overall and cause-specific excess of mortality in HIV-positive patients from 2004 to 2012 in the cohort of the Spanish Network of HIV Research (CoRIS), compared to Spanish general population and to assess the impact of multiple risk factors. We investigated differences between short-term and long-term risk factors effects on excess of mortality. Multiple Imputation by Chained Equations was used to deal with missing data.

**Results:**

In 9162 patients there were 363 deaths, 16.0% were non-AIDS malignancies, 10.5% liver and 0.3% cardiovascular related. Excess mortality was 1.20 deaths per 100 person years (py) for all-cause mortality, 0.16 for liver, 0.10 for non-AIDS malignancies and 0.03 for cardiovascular. Short-term (first-year follow-up) excess Hazard Ratio (eHR) for global mortality for baseline AIDS was 4.27 (95% CI 3.06–6.01) and 1.47 (95% CI 0.95–2.27) for HCV coinfection; long-term (subsequent follow-up) eHR for baseline AIDS was 0.88 (95% CI 0.58–1.35) and 4.48 (95% CI 2.71–7.42) for HCV coinfection. Lower CD4 count and higher viral load at entry, lower education, being male and over 50 years were predictors for overall excess mortality. Excess of liver mortality was higher in patients with CD4 counts at entry below 200 cells compared to those above 350 (eHR: 6.49, 95% CI 1.21–34.84) and in HCV-coinfected patients (eHR: 3.85, 95% CI 0.85– 17.37), although it was borderline significant. Patients over 50 years old (eHR: 5.55, 95%CI 2.4–12.85) and HCV coinfected (eHR: 5.81, 95% CI 2.6–13) showed a higher risk of non-AIDS malignancies mortality excess. Excess of cardiovascular mortality was related with HCV coinfection (eHR: 6.68, 95% CI 1.25–35.73).

**Conclusions:**

Our results show overall, liver, non-AIDS malignancies and cardiovascular excess of mortality associated with being HIV-positive, despite improvements in HIV disease management and antiretroviral therapies. Differential short-term and long-term effect of AIDS before entry and HCV coinfection was found for overall mortality.

